# Therapeutic potential of mesenchymal stem cell-derived exosomes for regenerative medicine applications

**DOI:** 10.1007/s10238-023-01282-z

**Published:** 2024-03-01

**Authors:** Szymon Roszkowski

**Affiliations:** Division of Biochemistry and Biogerontology, Collegium Medicum, Nicolaus Copernicus University, Debowa St. 3, 85-626 Bydgoszcz, Poland

**Keywords:** Mesenchymal stem cells, Exosomes, Extracellular vesicles, Tissue engineering, Regenerative medicine, Wound healing, Bone regeneration, Cartilage repair, Preclinical studies, Translational research

## Abstract

Mesenchymal stem cell-derived exosomes have emerged as a promising cell-free therapy for tissue engineering. Compared to intact stem cells, exosomes have advantages like low immunogenicity and ability to carry regenerative cargo. This review examined the potential of exosomes to treat defects in skin, bone and cartilage. In preclinical models, exosomes improved wound healing, stimulated bone regeneration, and enabled cartilage repair by transferring proteins, mRNAs and microRNAs. Their effects were elicited by modulating inflammation, angiogenesis, cell proliferation and matrix synthesis. Exosomes represent a promising cell-free therapy for tissue engineering. However, challenges remain regarding scalable isolation, elucidating mechanisms, and translating this approach to human trials. Understanding these challenges will enable the successful clinical translation of exosomes for regenerative medicine applications.

## Introduction

Tissue damage from trauma, disease, aging, or congenital defects presents a major challenge in healthcare. An emerging field called regenerative medicine aims to address this by repairing, replacing, or regenerating damaged tissues and organs. Stem cells sit at the core of many regenerative strategies due to their capacity to self-renew and differentiate into specialized cell types. Of the various stem cell populations, mesenchymal stem cells (MSCs) have emerged as a leading candidate for cell-based therapies. MSCs possess multipotency, allowing their differentiation into mesodermal lineages like bone, fat, and cartilage. Additionally, MSCs demonstrate immunomodulatory effects by suppressing inflammatory responses and releasing trophic factors that stimulate tissue repair [1, 2].

However, clinical use of MSC therapies faces some risks. Intravenous infusion of MSCs has been associated with infusional toxicity. Ectopic tissue formation and tumorigenesis are also concerns if proliferation and differentiation become dysregulated [3, 4]. As an alternative approach, the use of extracellular vesicles (EVs) derived from MSCs is gaining increasing attention. MSCs release EVs such as exosomes and microvesicles, which mediate intercellular communication and mirror many of the therapeutic effects of MSCs themselves. Compared to cell-based treatments, EVs avoid risks like tumor formation [5].

MSC-derived EVs promote tissue repair through several mechanisms. They can transfer regenerative factors and genetic material to recipient cells, reprogramming their behavior. EVs also modulate immune responses and inflammation to create a pro-regenerative microenvironment. Additionally, EVs stimulate resident stem and progenitor cells to enhance endogenous repair processes [6, 7].

Numerous preclinical studies have shown promising results using MSC-derived EVs to treat skin, bone, and cartilage defects. In models of wound healing, EVs accelerated re-epithelialization, angiogenesis, and collagen remodeling [8]. For bone repair, EVs promoted osteoblast proliferation, differentiation, and mineralization [9]. MSC exosomes enabled cartilage repair by enhancing chondrocyte proliferation and matrix synthesis and by modulating inflammatory pathways. Across these tissues, exosomes elicited regenerative effects by transferring proteins, mRNAs and microRNAs.

This review highlights preclinical animal studies demonstrating the tissue regenerative effects of MSC exosomes. However, challenges remain regarding scalable exosome isolation, customization of exosome cargo, elucidating mechanisms of action, and translating this therapeutic approach to human clinical trials. Understanding the challenges in this field will open up new possibilities for the successful translation of exosomes into clinical applications. Overall, MSC-derived exosomes represent.

a promising cell-free therapy for tissue engineering that warrants further investigation.

## MSC-derived EVs/exosomes

Extracellular vesicles (EVs), a general term encompassing various molecules including exosomes, microvesicles, microparticles, ectosomes, oncosomes, apoptotic bodies etc., are naturally released from all cell types [10]. They are enclosed by a lipid bilayer and cannot replicate [11]. EVs derived from MSCs, including exosomes and shed microvesicles (50–1000 nm diameter), can mimic the biological activity of MSCs by horizontally transferring many functional molecules including mRNAs, miRNAs, proteins and lipids to the cellular microenvironment and target cells, and then mediate restoration of homeostasis and tissue regeneration through various mechanisms [12]. Small EVs (sEVs, diameter 50–200 nm) released from MSCs have emerged as promising therapeutic agents in a wide range of preclinical models. They are considered as major mediators of MSC therapeutic functions [13].

Exosomes are nano-sized membranous vesicles secreted from cell membranes with 5’-nucleotidase activity and physiological function. They are distinguished from other EVs, i.e., shed microvesicles (or ectosomes; 100–1000 nm) and apoptotic bodies (released during apoptosis; 1–5 μm) based on their size, origin (endosomal or plasma membrane), surface markers and cargo [14, 15]. Specifically, exosomes are defined as sEVs of diameter 40–100 nm, density 1.13–1.19 g/ml in sucrose gradient and “cup” or “dish” shaped morphology by electron microscopy analysis. They are released from most cells into the extracellular space after fusion of multivesicular bodies (MVBs) and the plasma membrane [16]. Exosomes are characterized by surface markers (CD9, CD63, CD81) from the tetraspanin family, heat shock proteins (Hsp60, Hsp70, Hsp90), membrane transport proteins (Rab GTPases, annexins), biogenesis-related proteins (ESCRT family, Alix, TSG101), metabolic enzymes (GAPDH, ATPase, PGK1) as well as cytoskeletal proteins, and also lipid rafts [17].

**Fig. 1:**
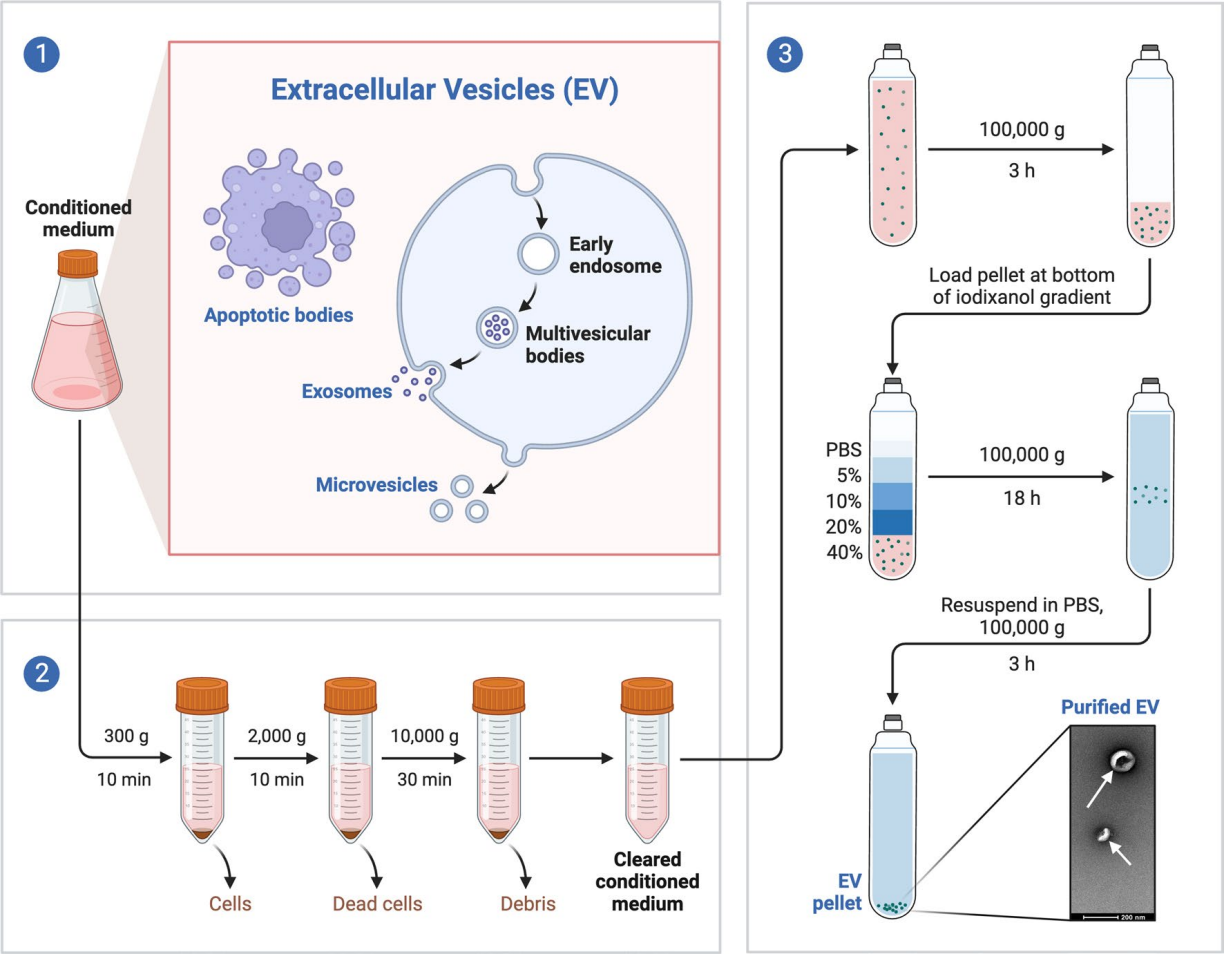
(1) Extracellular Vesicles (EV) are released by cells during their normal activity. (2) For EV separation, conditioned culture medium is harvested and major contaminants removed by consecutive low speed centrifugations. (3) The cleared supernatant is concentrated by ultracentrifugation, and the resulting 100K pellet is loaded on the bottom of an iodixanol gradient. EVs float upwards and EV-enriched fractions are collected and pelleted. The final sample is rich in EVs and absent in contaminants

Exosome isolation relies on various methods, each exploiting a particular exosome feature such as density, shape, size and surface proteins. These techniques include techniques based on differential ultracentrifugation, size, immunoaffinity capture, exosome precipitation and microfluidics techniques; however, ultracentrifugation is considered the gold standard method of exosome isolation. Figure 1 shows extracellular vesicle separation by density gradient ultracentrifugation [18].

Exosomes are released from most cells including MSCs and are present in all biofluids such as blood, urine, saliva, synovial fluid, bile, breast milk, amniotic fluid and semen [19]. Their cargo repertoire can reflect the nature and status of parent cells [13].

MSC-derived exosomes, like exosomes from other cell types, engage in intercellular communication and cell signaling by transferring proteins, mRNAs and regulatory microRNAs (miRNAs), as well as other paracrine mediators like cytokines and growth factors, to recipient cells. Hence, they can mimic and replicate MSC biological functions and can serve as safer, MSC-based, cell-free therapeutic approaches [20]. Therapeutic potential of exosomes released from MSCs derived from various tissues like bone marrow, adipose tissue, umbilical cord or placenta has been demonstrated in various disorders [13]. Here, we will focus on skin, bone and cartilage defects.

## MSC exosomes in skin wound healing

Chronic skin wounds represent a major clinical challenge, with substantial impacts on patient health and healthcare costs. Novel regenerative strategies using mesenchymal stem cells (MSCs) or their secreted exosomes show promise in facilitating wound healing through various mechanisms [21, 22].

MSC-derived exosomes can modulate inflammatory responses in chronic wounds by transferring regulatory miRNAs to target cells. For example, they can encourage anti-inflammatory M2 macrophage polarization and suppress pro-inflammatory M1 macrophages by shuttling miRNAs like miR-21, miR-146a, and miR-181 to modulate downstream inflammatory genes [6, 7]. This helps resolve prolonged inflammation that prevents healing. One study pretreated MSCs with melatonin to enhance exosome function before collecting and applying the exosomes to diabetic rat wound models [23]. Melatonin-enhanced MSC exosomes strongly suppressed inflammatory cytokines like IL-1β and TNF-α and promoted the anti-inflammatory factor IL-10 compared to non-pretreated exosomes.

Re-epithelialization, driven by epithelial cell proliferation and migration, is another key healing process impaired in chronic wounds that MSC exosomes can stimulate. One experiment applied umbilical cord MSC-derived exosomes to burn wounds in a rat model and found significantly accelerated re-epithelialization and cell proliferation compared to controls [8]. The exosomes activated Wnt/β-catenin signaling and upregulated proliferation/migration markers like CK19 and PCNA. They also inhibited apoptosis under heat stress by activating AKT signaling. In another study, the exosomes suppressed nuclear translocation of pro-apoptotic factors like AIF while upregulating survival proteins like PARP-1 to encourage re-epithelialization [24]. Engineering the exosomes to overexpress specific miRNAs like miR-135a can further enhance their epithelialization effects [25].

Recent work has combined MSC exosomes with biomaterial scaffolds to achieve optimal wound healing results. For example, a chitosan/silk hydrogel sponge loaded with exosomes from gingival MSCs markedly improved re-epithelialization, collagen deposition, angiogenesis, and neurite growth when applied to wounds in a diabetic rat model [26]. Another group loaded umbilical cord MSC exosomes into a Pluronic F-127 hydrogel which extended their release and activity at the wound site, boosting angiogenesis and wound closure rates [23].

Beyond modulating inflammation and epithelialization, MSC exosomes can also enhance two other critical phases of healing: angiogenesis and collagen/ECM remodeling. Multiple studies confirm exosomes derived from induced pluripotent, adipose, or other MSC sources stimulate collagen synthesis and angiogenesis when administered to cutaneous wound models [6, 27]. Specific exosomal miRNAs like miR-125a and lncRNAs like MALAT1 were implicated in inhibiting anti-angiogenic factors and encouraging these repair processes [25, 27].

A recent study by Hoang et al. investigated the regenerative potential of exosomes from various mesenchymal stem cell (MSC) sources on cutaneous wound healing [28]. The researchers isolated exosomes from adipose-derived MSCs (ADMSCs), bone marrow-derived MSCs (BMSCs), and umbilical cord MSCs (UCMSCs) and analyzed their growth factor content. Enzyme-linked immunosorbent assays identified differing levels of factors including platelet-derived growth factor BB (PDGF-BB), fibroblast growth factor 2 (FGF-2), vascular endothelial growth factor A (VEGF-A), and hepatocyte growth factor (HGF) in exosomes from all three MSC types. Notably, transforming growth factor beta (TGF-β) was only detected in exosomes from UCMSCs. Functional assays revealed that exosomes from each source induced proliferation and migration of both dermal fibroblasts and keratinocytes, indicating wound healing promotion potential. However, BMSC-derived exosomes exerted the greatest proliferative and migratory influence specifically on fibroblasts. In contrast, UCMSC-derived exosomes had the strongest effect on keratinocytes. Furthermore, higher doses of exosomes elicited faster cell migration rates overall. In summary, Hoang et al. demonstrated that while exosomes from various MSC origins share regenerative bioactivity, those derived from BMSCs and UCMSCs may be optimal for targeting dermal and epidermal cell populations in cutaneous wound healing. Their distinct growth factor content likely contributes to this source-dependent activity. These findings could inform the development of exosome-based therapeutics tailored to particular wound healing phases and cell types.

Collectively, these findings demonstrate the promise of engineered MSC exosomes, especially when combined with biomaterials, to promote comprehensive regeneration in impaired wounds. Further optimization of exosome production, cargo, and carriers could enable effective clinical translation.

Table 1 shows preclinical studies of mesenchymal stem cell-exosomes in cutaneous wound healing phases.

**Table 1:** Preclinical studies of mesenchymal stem cell-exosomes in cutaneous wound healing phases [29]

Wound healing phase	Exosome cellular origin	Model	Functional effects	Pathways	Refs
Inflammation	LPS-pretreated hUCMSCs	Rat diabetic cutaneous wound	M2 polarization	Let-7b via TLR4/NF-κB/STAT3/AKT	30
	hBMMSCs; hJMMSCs	Mice dorsal skin defects	Macrophage M2 polarization	miR-223 via pknox1	31
	hUCMSCs	Rat severe burn	M2 polarization. Inflammation alleviation	miR-181c via TLR4	32
	mBMMSCs	mBMMSCs	Promote beneficial regulatory T cell responses and M2 polarization	M2/Th2/Treg responses	33
Proliferation	hiPSC-MSCs	Rat dorsal skin wound	Accelerate skin cell proliferation and migration; promote collagen synthesis and angiogenesis	ERK1/2	7
	hUCMSCs	Rat skin burn	Enhance re-epithelialization and cell proliferation; reduce heat stress-induced apoptosis	Wnt/β-catenin; AKT	6
	hADMSCs	Mice full-thickness incision wound	remote fibroblast proliferation and migration; optimize collagen deposition	PI3K/Akt	34
Remodeling	hADMSCs	Mice skin incisional wound	Mitigating scar formation; promote ECM reconstruction	ERK/MAPK	35
	hUCMSCs	Mice full-thickness skin defects	Suppress myofibroblast differentiation and scar formation	TGF-β/SMAD2	25
	hAFSCs	Rat full-thickness skin wound	Anti-fibrotic scarring; suppress the excessive aggregation of myofibroblasts and ECM	TGF-β	36

*BMMSCs* Bone marrow MSCs, *ECM* Extracellular matrix, *hADMSCs* Human adipose-derived MSCs, *hAFSCs* Human amniotic fluid stem cells, *hBMMSCs* Human BMMSCs, *hiPSC-MSCs* Human induced pluripotent stem cell-derived MSCs, *hJMMSCs* Human jawbone marrow MSCs, *hUCMSCs* Human umbilical cord MSCs, *LPS* Lipopolysaccharide, *mBMMSCs* Mice BMMSCs, *MSCs* Mesenchymal stem cells, *TGF-β1* Transforming growth factor-β1, *TLR4* Toll-like receptor 4

## MSC exosomes in the regeneration of bone defects

Bone defects caused by trauma or disease can be treated surgically using bone grafts. Autografts are considered the gold standard due to their osteoinductive and osteoconductive properties, although they carry infection risk [37–39]. Synthetic scaffolds combined with mesenchymal stem cells (MSCs) are a promising alternative [40].

Despite effectiveness of MSC-based tissue engineering for bone regeneration, limitations exist including phenotypic changes during culture, low cell delivery and survival. This has driven interest in exploring cell-free alternatives using exosomes—extracellular vesicles secreted by cells [41].

Exosomes from bone tissue cells (e.g., MSCs, osteoblasts) regulate osteogenesis and bone remodeling. They induce osteogenic differentiation of MSCs by transferring specific miRNAs that modulate target gene expression [42, 43]. Exosomal miRNAs also stimulate osteoblast proliferation and angiogenesis [44]. For example, hypoxic preconditioning of MSCs increases exosomal HIF-1α, enhancing bone regeneration and angiogenesis in a rabbit osteonecrosis model [45].

Immobilizing MSC-derived exosomes on scaffolds enables sustained release, promoting bone regeneration through osteoinduction and MSC recruitment [46]. Combining iPSC-MSC-exosomes with β-TCP scaffolds boosted bone repair in a rat critical defect model by stimulating angiogenesis and osteogenesis [47]. iPSC-MSC-exosomes also prevented steroid-induced bone loss and increase local angiogenesis, probably through activation of the PI3K/Akt pathway in endothelial cells [48].

Additionally, MSC-exosomes have immunomodulatory effects that likely contribute to fracture healing by inhibiting the pro-inflammatory factors TNF-α and IL-1β and increasing the anti-inflammatory factor TGF-β [49].

In summary, substantial evidence indicates exosomes derived from bone tissue cells stimulate osteogenesis, angiogenesis, and bone remodeling to enhance bone defect repair through cell-free approaches. However, therapeutic efficacy may depend on health status of exosome donor cells.

As a recent study shows, BMSC-EXs obtained from type 1 diabetes rat models (dBMSC-EXs) and BMSC-EXs obtained from normal rats (nBMSC-EXs) both promoted the osteogenic differentiation of MSCs and increased the angiogenic activity of endothelial cells, but the therapeutic effect of dBMSCs-EXs was lower than nBMSC-EXs. These results indicate that autologous transplantation of exosomes from donors with chronic underlying diseases may be inappropriate for therapeutic purposes [50].

## MSC exosomes in the regeneration of cartilage defects

Cartilage defects arising from injury or osteoarthritis pose a major clinical challenge due to the poor intrinsic capacity of cartilage for self-repair. The avascular and aneural nature of cartilage means damage is not repaired via the typical wound healing mechanisms of most tissues. Furthermore, the low cellularity and lack of stem cell pools in cartilage mean the intrinsic regenerative capacity is limited [51, 52]. Current treatment approaches such as microfracture and autologous chondrocyte implantation have shown some success but are not optimal solutions [53]. Tissue engineering strategies using adult mesenchymal stem cells (MSCs) have emerged as a promising alternative cell source for cartilage repair [54]. MSCs can be isolated from various tissues including bone marrow, adipose tissue, synovium, and others [55]. These cells have chondrogenic potential and secrete tropic factors that can stimulate tissue repair. However, challenges remain with ensuring sufficient cell numbers, retention, and optimal differentiation at defect sites [56].

Recent studies have explored the use of MSC Exosomes as a cell-free therapeutic approach. MSC exosomes have shown preclinical efficacy in stimulating cartilage repair and regeneration through several mechanisms. Firstly, they can enhance chondrocyte migration, proliferation, and matrix synthesis to directly regenerate cartilage [57]. Secondly, they can inhibit inflammatory responses and apoptosis in osteoarthritic chondrocytes [58]. These effects are mediated through specific exosomal cargoes such as microRNAs (miR-140-5p, miR-92a-3p, miR-100-5p) and proteins (TSG-6, PTEN) that modulate signaling pathways including mTOR, Wnt/β-catenin, STAT3, and NF-kB [59].

MSC exosomes have been explored as standalone injections or in combination with biomaterial scaffolds to improve localization and retention [60, 61]. Scaffolds such as hydrogels and 3D-printed matrices provide structural support and can mimic the native cartilage extracellular matrix environment. Sustained release of exosomes from these scaffolds may be beneficial to exert biological effects over longer time periods to achieve functional cartilage repair [62].

## Problems with scalability of exosomes

One key challenge is scaling up isolation methods to obtain sufficient exosome quantities for clinical applications. Current benchtop techniques like ultracentrifugation and density gradients have limitations in exosome purity, integrity, and yields beyond small-scale research use [63]. For example, lengthy ultracentrifugation can damage exosomes through high g-forces [64]. Immunoaffinity methods using antibodies to capture exosomes are expensive and suffer from low yields [65]. New scalable methods are needed, such as tangential flow filtration [66], polymeric precipitation [67], microfluidics [68], and hydrostatic dialysis [69]. These can isolate bulk exosomes under mild conditions while maintaining integrity. However, further optimization is required to improve purity and prevent co-isolation of non-exosomal particles [70]. Standardization of techniques suitable for good manufacturing practices is also important for clinical translation [71]. Overall, innovative engineering solutions are still needed to enable scalable, reproducible exosome isolation that retains therapeutic activity.

## The benefits of customizing exosome cargo

Exosomes contain various bioactive molecules including proteins, lipids, mRNAs, and microRNAs that contribute to their biological effects. Emerging evidence suggests we can engineer exosomes with specific cargoes to achieve desired outcomes for different regenerative applications.

For instance, overexpressing particular microRNAs in parent mesenchymal stem cells can load enhanced levels of those miRNAs into secreted exosomes [72]. This allowed exosomes to better suppress tumor growth and angiogenesis when engineered with miR-146b [73], or to improve cardiac repair when engineered with miR-21 [74]. Likewise, treating parent cells with drugs or growth factors pre-conditions exosomes with higher concentrations of those agents for more potent effects [75].

Additionally, direct engineering methods to load exogenous cargo like siRNAs, mRNAs, and even drugs are being developed using electroporation, sonication, extrusion, and synthetic liposomes [76]. These customized exosomes retained functionality and successfully delivered cargo to recipient cells.

In summary, the capability to tailor exosome contents for specific therapeutic molecules or miRNAs holds great promise to enhance their natural regenerative properties. This could potentiate their effectiveness for applications in tissue engineering, drug delivery, and more.

## Engineered exosomes loaded with specific cargo

Several recent studies have engineered exosomes with particular proteins, mRNAs, or microRNAs as designer nanotherapeutics. For example, MSCs overexpressing miR-375 were shown to package the miRNA into secreted exosomes, which improved their efficacy in cardiac repair models [77]. In another study, MSC exosomes were loaded with exogenous siRNA against a target gene using electroporation, leading to successful siRNA delivery and gene silencing [78].

Additionally, directly packaging chemotherapeutic drugs into tumor cell-derived exosomes allowed targeted drug delivery to cancer cells in vivo [79]. The exosomes provided a protective, natural delivery vehicle. In a tissue engineering application, hypoxic culture conditions increased growth factor levels like VEGF in MSC-exosomes, enhancing their angiogenesis-promoting capacity [80].

These examples demonstrate the potential of developing engineered designer exosomes as advanced regenerative therapies. Loading exosomes with specific therapeutic cargoes could amplify their natural bioactive properties. Further optimization of cargo packaging approaches will support clinical translation of this innovative technology. Discussion of these engineered exosome studies provides important context on the future directions and possibilities in this rapidly evolving field.

## Exosome storage

Recent studies have shown lyophilization (freeze-drying) can significantly extend exosome shelf life compared to liquid storage. For example, lyophilized MSC exosomes retained structural integrity and biological activity for at least 6 months when stored at -20°C [81]. Refrigerated storage at 4°C in PBS maintains exosome quality for up to 7 days, while freezing at -20°C or -80°C can preserve intact exosomes for at least 90 days [82]. In contrast, living MSC potency declines within 1–3 days after refrigeration and 7–14 days frozen [83].

Additionally, exosomes tolerate freeze–thaw cycles better than cells. Repeated thawing and re-freezing decreased exosome particle count by just 13% compared to 61% loss of cell viability [84]. These emerging stability studies demonstrate the promise of exosome therapies to overcome limitations in cell product storage and shelf life. We can incorporate this data to highlight the improved stability of exosomes versus cells.

## Conclusions

Mesenchymal stem cell (MSC) derived exosomes have emerged as a promising new paradigm for regenerative medicine. Extensive preclinical evidence demonstrates their ability to enhance tissue repair and regeneration across diverse models of skin, bone, and cartilage defects. The therapeutic effects are mediated through transfer of bioactive proteins, mRNAs, and miRNAs that modulate cellular behaviors and microenvironmental factors to stimulate healing processes.

Exosomes overcome limitations of cell-based therapies, providing similar benefits in a safer, more stable extracellular vesicle format. They appear inherently immunomodulatory and lack risks like ectopic tissue formation. Exosomes can be isolated from various MSC sources such as bone marrow, adipose, and synovium. Their cargo can also be engineered for enhanced regenerative bioactivity.

However, challenges remain in translating these preclinical findings to clinical applications. Further optimization is needed in scalable production, cargo enhancement, controlled biodistribution and release kinetics, as well as mechanistic understanding. Combining exosomes with biomaterial scaffolds appears promising to improve localization and retention. Larger studies directly comparing exosomes to native MSCs will better define their advantages.

Overall, MSC-derived exosomes represent a novel nanotherapeutic strategy with significant potential but remains early in the developmental pipeline. Continued multidisciplinary research to address translational barriers will accelerate the impact of this innovative nanomedicine approach on regenerative therapies.

## Data Availability

Not applicable.
